# Influence of Drying Techniques on the Physicochemical, Nutritional, and Morphological Properties of Bighead Carp (*Hypophthalmichthys nobilis*) Fillets

**DOI:** 10.3390/foods10112837

**Published:** 2021-11-17

**Authors:** Kamal Alahmad, Wenshui Xia, Qixing Jiang, Yanshun Xu

**Affiliations:** 1 State Key Laboratory of Food Science and Technology, School of Food Science and Technology, Collaborative Innovation Center of Food Safety and Quality Control in Jiangsu Province, Jiangnan University, Wuxi 214122, China; kamalalani85@yahoo.com (K.A.); qixingj@163.com (Q.J.); xuys@jiangnan.edu.cn (Y.X.); 2 Department of Food Science and Technology, Faculty of Agriculture, University of Alfurat, Deir Ezzor, Syria

**Keywords:** bighead carp (*Hypophthalmichthys nobilis*), volatile compounds, HPLC, vitamins, DSC thermal stability, fatty acids

## Abstract

Different experiment analyses were performed to evaluate the influence of two drying techniques (oven drying and microwave drying) on the fillets of bighead carp fish (*Hypophthalmichthys nobilis*). The processed and fresh samples were subjected to the chemical analysis of (amino acids, minerals, volatile compounds, fatty acids, and vitamins) as well as scanning electron microscopy, thermal analysis, and color measurement, in order to identify nutritional components that can be additives or supplementary in food industries. The drying techniques increased the protein content significantly. Amino acids were identified, and the level of essential amino acid (EAA) was higher under the microwave treatment compared with the oven drying process. The Ca^+2^ and K^+1^ were presented in high values, followed by Na^+1^ and Mg^+2^. In addition, the drying techniques showed and released more volatile compounds in the processed samples compared with the unprocessed samples. Under the drying process, polyunsaturated fatty acids were increased in the processed fillets, whereas the level of saturated and monounsaturated fatty acids reduced. Thermal degradation occurred from 100 to 150 °C. However, the processed samples were subjected to an intensive endothermic response, but remained stable until 100 °C. Therefore, the microwave technique showed some enhancements in the nutritional value and has the potential to be applied as an effective preservation method of bighead carp fish. Furthermore, dried fillets could be an alternative source of bighead carp fish for the food industry.

## 1. Introduction

Bighead carp (*Hypophthalmichthys nobilis*) is found in freshwater regions, vast lakes, and rivers worldwide [1]. These species can survive in huge different freshwater environments, promoting the ability to live in different parts of the globe. Fish is a huge source of protein due to its enriched nutritional values, good protein quality containing efficient amounts of amino acids, considerable amounts of fatty acids, and abundant vitamins, such as riboflavin (B2) and pyridoxine (B6), cyanocobalamin (B12), and D3. Carp fish spawn several times during one season, and the water temperature of 20 °C is suitable for the start of spawning [2,3]. Bighead carp fish prefers a water temperature range of 4 to 26 °C and originally inhabits Asia, particularly China and southeastern China [4]. The fish can survive in many countries in other parts of Asia, such as Thailand, Myanmar, and Vietnam, as well as in Europe and Northern America. Freshwater fish including silver carp, grass carp, and bighead carp contains up to 75% of moisture and is greatly spoiled and damaged, during its short storage life. The musty odor and taste in fresh fish can reduce the consumption of fish products. However, to improve the quality of fish preservation and make the final product acceptable by consumers, then some preservation processing techniques should be applied.

Drying of fish is important, since it preserves fish by inactivating enzymes and removing the high amount of moisture which can stop bacterial and mold growth, and prevent the spoilage of fish products [5,6]. The drying techniques can be generally classified as thermal drying, osmatic dehydration, and mechanical removing of moisture. Fish can be dried or smoked, which can extend the shelf life, improve the flavor and texture, as well as make the final product more preferable for consumption. Drying is a technique of simultaneous heat and mass transfer. The heat causes evaporation of water from the surface and mass transfer of water from the interior to the surface of the fish. Water evaporation takes place due to the vapor pressure difference between the fish and the surrounding environment [7]. Fish drying methods vary among and within countries depending on the species used and the type of product desired. Fish may be dried to various degrees with the water content in the final product ranging from about 10% to 30%.

Different fish species have different protein contents, ranging from 8% to 22% and leading to 60–80% of energy produced in various carp fish species [8]. Researchers detected different water- and fat-soluble vitamins in freshwater fish or smoked fish, which were presented without significant differences in the treated samples [9]. The vitamin C content in fish fillet is 4.7–6.5 mg/100 g. For the same fish species, the contents of B3 and B2 in the smoked samples are 5.4 and 0.42 mg/100 g fillet, respectively [9]. However, the drying process is important to stop or reduce undesirable changes in fish species. The traditional drying techniques include solar drying, hot air drying, and smoking [10]. In addition, people around the world use different techniques of preservation, such as heating, smoking, salt curing or fermentation. The dried fish product is a tasty and suitable food source and can be used in various food applications. In ancient times, solar drying is a traditional and cheap method of preserving fish [11]. The second technique is the hot air drying process, which can control the air velocity, humidity, and experimental conditions. Another advantage of this technique is the flexibility of loading fish samples in the oven. With the efficient use of energy, the fish product can be improved during the drying process and the quality changes potentially [12]. The microwave technique with high heating energy efficiency is safe, harmless, and easy to control. Microwaves heat materials simultaneously from the inside and outside, which can apply high rates of evaporation. Therefore, it is an efficient process for meat and fish, since it needs less time and energy and improves the final product with nutritive quality [13]. Different parameters, such as the temperature, power or sample shape, can be monitored during the drying process [14].

Less data have been published on the effect of drying techniques on the volatile compounds, as well as thermal and morphological characteristics of bighead carp. Therefore, in this study, we investigated the influence of two drying techniques (oven drying and microwave drying) on the nutritional (amino acids, minerals, volatile compounds, fatty acids), morphological, and physicochemical properties of bighead carp (*Hypophthalmichthys nobilis*) fillets.

## 2. Materials and Methods

### 2.1. Materials

Fresh bighead carp fish (3 to 5 kg, 40 to 50 cm length, between 3 to 4 years of age) was purchased. To ensure the quality and freshness of the fish, fresh bighead carp (Hypophthalmichthys nobilis) was obtained from a research center (Yangtze River aquatic products science and technology industry Co., Ltd. in Wuxi, Jiangsu, China). The fish was caught from one location and handled according to the standard procedures for storage and transportation (washing, aligning in the tub, and icing). In addition, the samples were received within 24 h and the freshness of the fish was checked (skin, eyes, and gills), to ensure that it was free of mechanical injuries and did not change its color, smell, and texture. In addition, the similar-sized fish was selected for sampling, and the samples were mixed well to form a homogeneous sample. The solutions, acids, and alkaloids were purchased from Sinopharm Chemical Reagents Co., Ltd. (Shanghai, China). The other reagents and chemicals used were of analytical grade.

### 2.2. Sample Preparation

The targeted fish was cleaned with tap water, and the head and bones were removed. The flesh was chopped into small slices of 6 to 7 cm length, 3 to 4 cm width, and 1.5 to 2 cm thickness, to obtain the fish fillet. The raw samples were ground, homogenized, and remained frozen at −20 °C until further experiments. Drying was carried out using two different methods. The first portion of the sample was dried using a laboratory hot air-drying oven with a temperature range of 50–300 °C (Oven DHG-9030A: 490 × 500 × 625 mm, Suzhou Qiandong, Suzhou, China) at 90 °C for 150 min. The second portion of the sample was dried using a microwave oven (ORW1.0S-5Z, 100–1000 W, 380 V, 40 × 37 × 22 cm/Nanjing Aorun Microwave Technology Co., Ltd, Nanjing, China.) according to Wu and Mao [15]. With some modifications, the sample dried at 2450 MHz and 360 W for 10 min. The dried bighead carp fish fillets were then blended and homogenized using a high-speed blender (25,000 rpm).

### 2.3. Chemical Composition Analysis

The raw and dried samples were analyzed individually. The contents of protein, fat, moisture, and ash were determined using the AOAC standard methods 925.09, 932.06, 985.2912, and 923.03, respectively [16]. Briefly, 3 g of each sample was weighed in a furnace crucible and burned at 550 to 600 °C for 5 h in a muffle furnace until a constant weight was obtained and the color changed to reddish ash. The lipid content was estimated by extracting 3 g of the sample in a solvent (petroleum ether) in Soxhlet extraction instrument (Auto fat determiner, Xian Jian Instruments Co., Shanghai, China) for 4.5 h. Kjeldahl analyzer (DK-3400/FOSS, Hilleroed, Denmark) was used to evaluate the crude protein of fish samples by multiplying the determined total nitrogen by 6.25 (standard factor). The moisture content was evaluated by drying 3 g of the sample for 2 to 3 h at 105 °C until a constant weight.

### 2.4. Mineral Determination

The mineral content was determined using the method of Shahidi et al. [17] with slight modifications. Briefly, 1 g of each sample was weighed and burned at 550 °C in a muffle furnace (JNL-12XB; Luoyang Liyu Furnace Co Ltd., Luoyang, China). The obtained ash was dissolved in 5.0 mL of HNO_3_/HCL/H_2_O (mixed solution, 1:2:3) and then heated lightly on a hot plate until the gray-brown fumes faded. Around 5 mL of high-purity water (analytical grade) was added and the heating continued until the mixture became colorless. The solution of each sample was transferred into a separate volumetric flask (25 mL) utilizing Whatman No.45 filter paper, and the volume was made to the mark by adding the analytical grade water. The obtained solution was used to determine the concentrations of the minerals in the fish samples on Varian Spectra AA-220 FS Atomic Absorption Spectroscopy (Varian Inc., Australia). The following minerals (Na^+1^, Mg^+2^, K^+1^, Ca^+2^, Fe^+2^, Cu^+2^, and Zn^+2^) were detected and calculated as mg/100 g of the original weight of each sample.

### 2.5. Amino Acid Profile Determination

Briefly, 0.1 g of the raw and dried samples were digested with 6 M hydrochloride HCl for 24 h at 110 °C under nitrogen atmosphere in an air oven. After the hydrolysis, the samples were cooled, washed using pure water, filtered with Whatman filter paper, and centrifuged at 10,000 for 15 min. The amino acid components were separated and analyzed using high-performance liquid chromatography (HPLC, Agilent Technologies, model 1100, Santa Clara, CA, USA) under the reverse phase mode using (180 mm × 4.6, Agilent) Zorbax 80 A with a C18 column at 40 °C and detection at 338 nm with a flow rate of 1 mL/min. All of the samples were analyzed in triplicate, and the amino acid composition was expressed as grams of amino acid per 100 g of the protein sample.

### 2.6. Determination of Volatile Components

The volatile components were analyzed using GC–MS [18]. The compounds were separated on a CP-Sil-8CB (Varian, Walnut Creek, CA, USA) combined with silica in the capillary column (30 m length, 0.25 mm, id, and 0.25 μm film thickness) in Varian (model 3800, Varian, Palo Alto, CA, USA) gas chromatography. The injection port was kept at 220 °C, the interface of flame ionization detector (FID) interface was preserved at 250 °C, and the compounds were achieved and separated on the column (30 × 0.25 μm, J and W Scientific, Folsom, CA, USA). The compound separation was programmed as follows: The temperature was set at 50 °C for 3 min, 80–100 °C for 5 °C/min, 100 –220 °C for 5 to 10 °C/min, and 230 °C for 5 min. Helium gas was used as the carrier gas, and the flow in the regular column was 0.8 mL/min. However, the injector was maintained at 240 °C. The parameters of GC–MS included the following: Ionization mode (EI+), described as emission current: 200 µA; electron energy: 70 eV; interface temperature: 250 °C; source temperature: 200 °C; and detector voltage: 350 V. The identification and separation of volatile components were considered based on retention indices and a comparison of the compounds in all of samples with standard compounds in Wiley library.

### 2.7. Fatty Acid (FA) Composition

Total fats were extracted from bighead carp fish fillets (1.0 g) hexane:methanol (2:1, *v/v*), then a small amount of ascorbic acid (0.05 mg/mL) was added to prevent autoxidation. The fatty acid composition was analyzed and investigated according to a previously reported method [19] with modifications. Briefly, 20–50 mg of fat was prepared by transesterification with the addition of methanol (5 mL) with 2% sulfuric acid, then heating the mixture in a water bath (50 °C) for 3 h. Thereafter, the fat was extracted using pertroleum ether (40 °C) and kept over anhydrous sodium sulfate. FA was analyzed under (GC-7820A–model Agilent, Santa Clara, CA, USA) conditions equipped with a flame ionization detector. The fatty acids were separated on a liquid capillary column (TRACETRFAME, 60 m × 0.25 mm × 0.25 µm Thermo Fisher, Waltham, MA, USA), with a carrier gas (nitrogen) under the flow rate of 1.2 mL/min. The starting column temperature remained at 120 °C for 2 min. Thereafter, the column was conducted at 190 °C for 3 min, before increasing to 220 °C for 10 min with a flow rate of 5 °C/min. The composition of fatty acids was identified by comparing retention times (RT) in (GC) peaks with 40 standards of fatty acid methyl esters. The measurement was conducted three times.

### 2.8. Vitamin Analysis

The water-soluble vitamins in fish samples were analyzed using a previously reported method [20] with modifications. Briefly, 5 mL of HPLC-grade water with 1 mL of hexane solvent were mixed with 0.5 g of bighead carp fish samples. The mixture was homogenized for 5 min and then left in a sonication machine for 20 min at 30 °C. The solution was centrifuged at 5000 rpm for 20 min and filtered through a filter paper (0.45 µm filter) before injection into the HPLC system. The filtered solution (10 µL) was injected into the HPLC system (model-Agilent 1100 Technologies, Santa Clara, CA, USA), column C18 (250 × 4.6 mm). The column was applied for the separation of water-soluble vitamins in the sample at room temperature under the flow rate of 1 mL/min. Approximately 0.5 g of fish samples were transferred to a 6 mL tube locked and sealed firmly to detect the D3 vitamin (a fat-soluble vitamin). Subsequently, 3 mL of 95% ethanol was added to 0.2 mL of 60% hydroxide potassium (KOH). Small amounts of pyrogallol and ascorbic acid were added to the mixture. The sample was appropriately closed and then saponified by heating to 60 °C for 15 min. The matter was extracted by adding 2 mL of purified water mixed with 2 mL of n-hexane. The sample was vortexed for 30 s. The top layer was filtered through (0.45 µm filter), and the filtered solution was injected into the HPLC system [21].

### 2.9. Scanning Electron Microscopy (SEM) Analysis

The SEM analysis was carried out using a scanning electron microscope instrument (HITACHI-High-Tech’s-SU1510-Minato-ku, Tokyo, Japan) to detect the morphological properties of the raw and dried samples (oven- and microwave-dried). Prior to loading the samples to the SEM instrument, the treated samples were coated. The image was viewed under 1.0 KV by a secondary electron image. The image was scanned using a 10.20 mm Ricoh Camera with 600× magnification.

### 2.10. Thermal Characterization Analysis

The fresh and dried fish samples were heated utilizing the differential scanning calorimeter instrument DSC (DSC-Q200-V24.8 Build 120, TA instrument, New Castle, DE, USA). The raw and dried fish samples (20 ± 1 mg) were completely tight and sealed in aluminum pans and then gradually heated in a DSC machine with a temperature range of −50 to 200 °C at a flow rate of 10 °C/min. Then, the sample was maintained for 7 min at 200 °C. The samples were cooled to −50 °C under the flow rate of 10 °C/min. An empty aluminum pan was utilized as a control or reference sample.

### 2.11. Color Measurement

The color of bighead carp samples was scaled utilizing a Hunter Lab digital colorimeter (TC-PIIG system; Beijing Optical Instrument Co. Ltd., Beijing, China). The treated samples were placed at the port of the instrument. L*, a*, and b* values of color evaluation were recorded. L* represents lightness; a* represents redness and b* represents yellowness (yellow/blue) [22]; and (ΔE) represents the total color difference. The following Equation (1) was used to calculate the (ΔE) index, as described by Abdalhai et al. [23]:

(1) $$∆E=\sqrt{\left({\Delta L}^{*2}+{\Delta a}^{*2}+{\Delta b}^{*2}\right)}$$

### 2.12. Statistical Analysis

All of the data were presented as the mean value ± standard deviation (±SD), and the samples were analyzed in triplicate (*n* = 3). The results were subjected to the one-way analysis of variance (ANOVA) to detect the significant differences among the samples. Duncan’s test was used to analyze multiple ranges between the means using SPSS version 19 (SPSS. Chicago, IL, USA). The significance level was defined at *p* ≤ 0.05.

## 3. Results and Discussion

### 3.1. Proximate Chemical Composition

**Figure 1:**
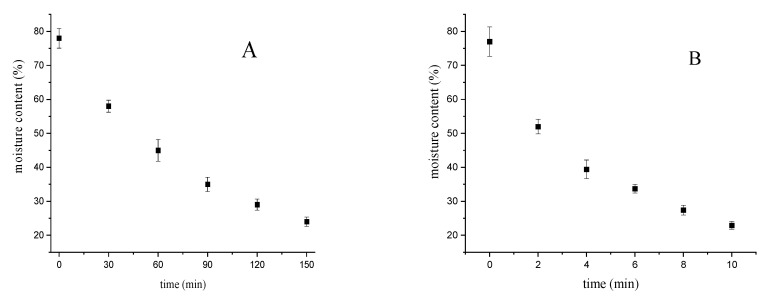
Drying curves of bighead carp fillets. (**A**) Hot air drying; (**B**) microwave drying.

The chemical composition of bighead carp fish under different drying techniques (oven drying and microwave drying) is shown in Table 1. The correlation curve between the drying time and moisture content of the processed fillets is given in Figure 1. The moisture content decreased with the increasing time of the hot air drying technique, which could be attributed to the increase in the evaporation of water on the surface of fish fillets. In addition, the evaporation speed decreased by the drying time associated with the end of the drying process. Moreover, the moisture content under the microwave technique decreased with the increasing drying time, which could be attributed to the rapid evaporation of moisture on and in the surface of fish fillets, due to increasing the drying temperature by microwave heating [24]. Therefore, the decrease in the moisture content was the most important change in the fish fillet after the drying techniques. In the raw fillets, the moisture values were 77.69%, and under the oven and microwave drying techniques the values were 24.19% and 23.92%, respectively. The high moisture content of fish products leads to an increase in the microbial and enzymatic decomposition of fish. Therefore, the drying techniques could reduce the moisture value and improve the quality of food products. No significant differences (*p* < 0.05) in fat values were obtained using the different drying methods. The increase of the protein content in the dried fillets compared with the raw fillets was recorded, indicating that the application of the drying techniques had no significant influence on the loss of amino acids. The protein values were not significantly different between the oven and microwave drying techniques based on the dry weight.

The distribution of fat values were 5.69%, 5.88%, and 5.93% dwt in processed and raw fillets, respectively. The ash contents were 4.83% dwt under oven drying and 4.84% dwt under microwave drying, while in the raw fillets the value was 4.26% dwt. The protein, ash, and fat contents increased in the processed fillets on a fresh weight basis. Moisture is an important parameter for food storage, and microbes start growing in food products with more than 12% of moisture [25].

Fats help the human body in the absorption of nutrients. In addition, they induce important compounds that provide our body with efficient energy and support cell growth in order for some functions to work regularly. The protein and fat contents increased significantly in the processed fillets based on the fresh weight basis (*p* < 0.05). The significant and remarkable increase of protein levels in dried fillets compared with the raw fillets suggested that the protein remained and was not lost during the drying process, which was similar to the previous studies related to the fish drying process [15,26]. The protein content increased from 17.72% in the raw fillets to 64.57% and 64.89%, respectively in the oven- and microwave-dried fillets. However, there were no significant differences between the microwave and oven drying techniques in the protein content. Protein, as the main chemical composition of fish carp and other fish species, had different values under the different drying techniques based on previous studies. The protein content recorded 69% using the oven drying technique [15].

### 3.2. Mineral Composition

The mineral results in the bighead carp samples were shown in Table 1. The function of major minerals is to regulate the body’s tissue growth in order to build strong bones. The sodium element Na^+1^ reported 647.85, 647.63, 648.57 mg/100 g dwt, respectively in microwave, oven, and raw fish fillets. Therefore, no significant differences were shown during the drying techniques compared with the raw fish fillet. However, based on a previous study, (Na^+^) recorded 210 mg/100 g under the oven treatment [27]. The content of Mg^+2^ in all of the treated samples was recorded without a significant difference. Calcium was recorded as 978.17 and 976.89 mg/100 g dwt, respectively under the oven and microwave techniques. Therefore, the drying techniques could somewhat reduce the content of calcium during the drying process compared with the raw fillets. Potassium (K^+^) showed 793.54 mg/100 g dwt in the raw fillet and 787.93 mg/100 g dwt using oven drying compared with 789.28 mg/100 g dwt under the microwave process. These values showed no remarkable influence on the content of total minerals during the drying techniques. Based on the previous studies, (K^+^) ranged from 230 to 250 mg/100 g using sun drying, while (Ca^+2^) ranged from 90 to 160 mg/100 g under the oven drying technique [27]. The iron content values showed a slight significant difference between the treated samples, which could be due to the fact that drying has no major influence on the content of (Fe^+2^).

Copper (Cu^+2^) and zinc (Zn^+2^) resulted in trace amounts. Generally, potassium is important for the healthy functioning of the heart and calcium is important for building bones and teeth [28]. Zinc and copper assist with the functioning of the nervous system, while sodium assists with the preservation of water balance in the human body [29]. The fish bighead carp based on the mineral analysis results showed that sufficient amounts of major minerals in the raw and processed samples are needed for our body to maintain healthy functioning along with enhancing our body’s health.

### 3.3. Amino Acid Composition

The amino acid concentrations are shown in Table 2. In the current study, the drying process caused significant changes in different amino acids between the raw and dried fillets. Methionine had a high value of 3.65 and 3.52 g/100 g dwt, respectively using the oven and microwave treatment. However, leucine had a value of 7.51 g/100 g dwt in the oven-dried fillets and 7.68 g/100 g dwt in the microwave-dried fillets. Table 2 shows that lysine was significantly (*p* < 0.05) higher in the microwave-dried fillets compared with the oven-dried fillets. Glutamic acid was reported to have a high value of 16.36 and 15.80 g/100 g dwt, respectively under oven and microwave drying. Histidine reported a value of 2.31 g/100 g dwt in the oven-dried fillets and 1.98 g/100 g dwt in the raw fillets. The oven treatment enhanced the histidine content during drying. Tao et al. reported the histidine values of 2.37 and 2.89 g/100 g dwt, respectively in grass carp in the hot air and microwave drying techniques [15]. Compared with the amino acid values in raw fillets, phenylalanine and isoleucine showed a significant loss after the drying process due to the heating treatment.

The major amino acids found in fish fillets were leucine, lysine, glutamic acid, and aspartic acid, while methionine, serine, proline, and cysteine were present in small quantities. According to the FAO/WHO/UNU amino acid recommendation, leucine was higher in microwave-dried fillets compared with the required dose of leucine for children and adults, respectively [30]. However, TAA was higher in microwave-dried fillets compared with oven-dried fillets. The current study observed that the bighead carp fish is an important and good source of amino acids. However, the loss of some amino acid contents suggested that the chemical reaction during the drying process showed a few changes were related to the thermostable property during the drying process. Generally, the high and sufficient amounts of amino acids play an important role in protein synthesis and the quality of the protein depends on the composition of amino acids. Therefore, the drying techniques could improve the content of essential amino acids compared with raw freshwater fish species.

### 3.4. Volatile Organic Compounds Analysis

As explained in Table 3, in this study, 23, 23, and 19 volatile compounds were identified in oven-, microwave-dried, and raw fillets, respectively. Hydrocarbon, aldehydes, alcohols, and ketones were observed in the current study. Abundant amounts of volatile compounds were found in the dried fillets. Hexanal was 1.01% and 1.13% in the oven- and microwave-dried fillets, but recorded 0.15% in the raw fillets. Alcohol (1-Penten-3-ol) showed different values that ranged from 0.69% to 0.96%. However, the heating treatment could enhance and release some volatile compounds. Nonanoic acid was found in the dried fillets, but was undetected in the raw sample. Additionally, due to the drying effect, which could influence the amount of volatile compounds, nonadecane was higher in the raw fillets compared with the dried fillets. A few aldehydes and alcohols compounds were higher in the dried fillets, but a few alkane carbonyl components were slightly higher in the raw fillets. Based on a previous study, some volatile compounds were higher in the cooked fish [31]. Acetic acid, 2-butanol, and 3-methyl were found in this study in different amounts compared with other studies that reported volatile compounds in smoke-dried fish [32].

### 3.5. Fatty Acid Analysis

#### 3.5.1. Saturated Fatty Acids (SFA)

In oven-dried and raw fillets, palmitic acid (C16:0) had the highest value of SFA (Table 4), with a significant difference (*p* < 0.05). However, myristic acid (C14:0) and lauric acid (C12:0) were decreased significantly during the drying process, which could be due to the heating treatment. Octadecanoic acid (C18:0) significantly decreased during the drying techniques (*p* < 0.05), and recorded (6.44%) in raw fillets compared with (4.9%) and (4.6%) in microwave- and oven-dried fillets, respectively. The sum of saturated fatty acids was decreased through the drying process from (32.5%) in raw fillets to (30.69%) in microwave-dried fillets and (29.37%) in oven-dried fillets. Drying could influence the ester bonds between the saturated fatty acids and lead to the reduction of the total quantity of SFA.

#### 3.5.2. Monounsaturated Fatty Acids (MUFA)

The processed fillet under the drying techniques observed different values of (MUFA). The raw and treated fillets showed the top values of (MUFA) in total fatty acids composition. Seven monounsaturated fatty acids were detected. However, oleic acid (C18:1) showed the highest value of fatty acids (Table 4). During oven and microwave drying, most of the monounsaturated fatty acids decreased significantly (*p* < 0.05). However, (C16:1) palmitoleic acid was found in insufficient amounts, and C22:1 and C24:1 were found in low amounts in oven-dried and raw fillets. The total content of MUFA was reduced from 40% in raw fillets to 34% in microwave-dried fillets and 37% in oven-dried fillets.

#### 3.5.3. Polyunsaturated Fatty Acid Composition

PUFAs were presented and ranged from (26.7%) in raw fillets to (32.85%) in dried fillets. Linoleic acid (C18:2 *n*-6) was the highest value among the polyunsaturated fatty acids in this study. The concentration of linoleic acid was (7.3%) in raw fillets compared with (8.27%) and (7.7%) in oven- and microwave-dried fillets, respectively. Linoleic acid was increased significantly in processed fillets. The α linolenic acid (C18:3 *n*-3) was reported in a high value after the drying techniques and significantly increased (*p* < 0.05). In addition, the content of raw, oven-, and microwave-dried fillets were 5.02%, 5.96%, and 5.30%, respectively.

Arachidonic acid (C20:4 *n*-6) was found in sufficient amounts in all of the fillets (raw and dried fillets) and varied from about (2.92%) in raw fillets to (4.27%) in microwave drying fillets. DHA and EPA [C20:5, C22:6] are naturally occurring and typically exist in fish products. Microwave-dried fillets presented the top value of EPA and DHA after drying followed by the oven-dried fillets. EPA values were (3.47%), (4.99%), and (5.29%) for the raw, oven-, and microwave-dried fillets, respectively.

This research showed that bighead carp fillets are a good source of fatty acids (SFA, MUFA, and PUFA), especially PUFA, which observed high amounts practically after drying. Fish is a good source of ω-3 and ω-6. Oleic acid (C18:1) was in a high amount in this study as compared with Tao Wu et al. [15].

PUFAs in the current study were 26% in raw fillets compared with 20% to 21% of the same carp reported by [33]. The immune system with chemical reactions and inflammation mechanisms can be affected by polyunsaturated fatty acids. Therefore, in fish food, ω-3 and ω-6 exist and have an important effect on the lipid structure of the cell membrane [33,34,35]. Overall, the ratio between (PUFA/SFA and MUFA/SFA) nutritionally could provide us the nutritional composition of lipid structure. Similar to the previous studies, some researchers found the ratio (PUFA/SFA and MUFA/SFA) effect on dietary fat and lipid levels in different parts of rats animal [36].

### 3.6. Vitamin Analysis

The vitamin analysis results of bighead carp fillets under the two drying methods are presented in Table 1. Therefore, vitamins (K, A, E, and D) exist in the flesh of aquatic fish, while water-soluble vitamins can be found in different fish. Naturally, water-soluble vitamins were classified under different types of vitamins, such as folic acid (B9), pantothenic acid (B5), thiamine (B1), riboflavin (B2), pyridoxamine (B6), and vitamin C. Generally, vitamins in both types (water-soluble and fat-soluble) play an important role in maintaining the body functions, such as digestion and metabolism. However, vitamins could also be antioxidants, which are very important for health and metabolism processes [22].

The water-soluble vitamin B complex and vitamin C are required in larger quantities and have functions as co-enzymes [37]. The vitamin C values in the dried fillets showed no significant variation (*p* ≤ 0.05). Thiamine (B1) was high in microwave-dried fillets compared with oven-dried fillets. However, this result was higher than the B1 values reported by Ersoy et al. [38].

In the metabolism of carbohydrates, thiamin functions as the co-enzyme co-carboxylase of thiamin pyrophosphate (TPP) and plays the key point of regulation in the TCA cycle [37]. Riboflavin (B2) was 0.24 mg/100 g dwt under the oven drying treatment compared with 0.27 mg/100 g dwt in microwave drying, while it reported 0.22 mg/100 g dwt in raw fillets. These values were higher compared with the values reported in [38]. In this study, cyanocobalamin (B12) ranged between 0.01 and 0.02 mg/100 g dwt. Vitamin (B12) is important for the maintenance of the nervous system functions and plays a role in maintaining red blood cells at a balanced level in order to protect the body from inflammations.

Vitamin D is a fat-soluble vitamin and showed no significant variation. However, the results were almost similar to [39]. Vitamin D exists in small amounts in fish products. In addition, as known, vitamin D is necessary for bone growth during childhood. Moreover, it could promote and improve mineral absorption, as well as reduce inflammations.

### 3.7. Scanning Electron Microscopy (SEM) Analysis

**Figure 2:**
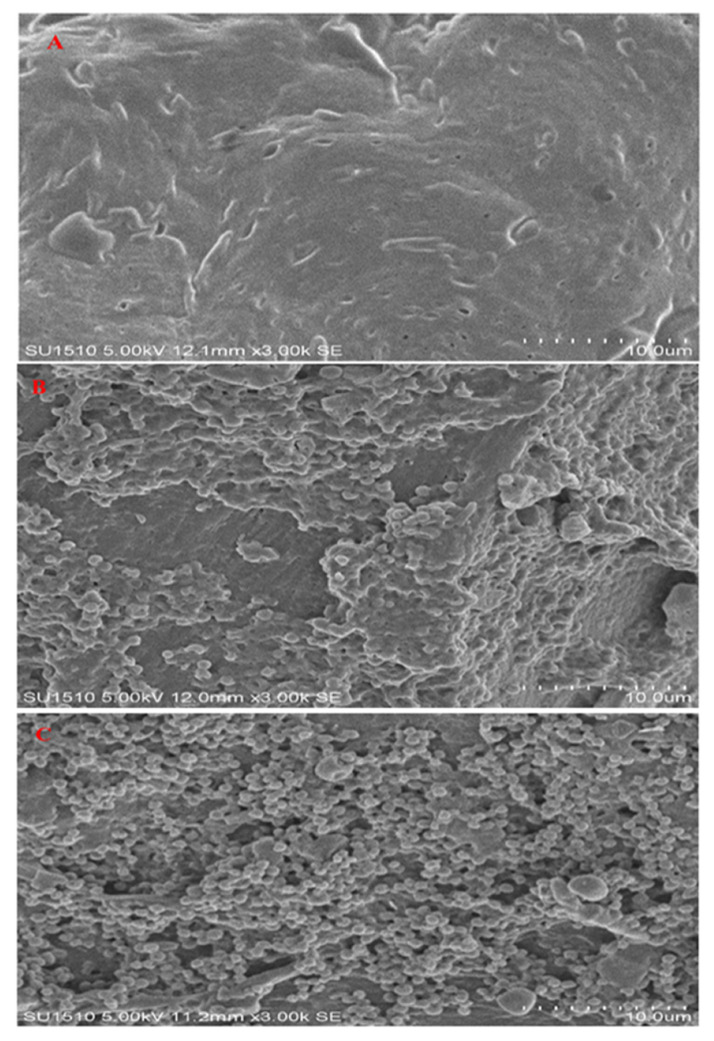
Scanning electron microscopy of bighead carp *H. nobilis* fillets. (**A**) Raw; (**B**) oven drying; (**C**) microwave drying.

The observations of the raw and treated fillets by SEM are shown in Figure 2. The surface of dried fillets (B, C) is highly deformed compared with the raw fillets (A). Under the SEM analysis, the surface of the samples showed significant variations in the size of particles and shapes. The surface of particles in raw fillets was clear and smooth without rough angles or folding. However, the drying process affected the aggregation of size particles in samples (B, C). Therefore, particle sizes showed differences due to the differences in the aggregation of protein particles.

In general, the observation from the SEM micrographs showed that some particles have a spherical shape, which could be attributed to the effect of water evaporation released from the treated samples and the moisture loss during the long drying process, which is similar to the result reported by Moradi et al. [40]. The processed fillets were significantly damaged and the texture of the fillets were rough and crumbled due to the potential of electromagnetic waves and increased temperature during the drying process. The high pressure inside the dried samples (B, C) due to the high vapor pressure during microwave irradiation and internal pressure can also accelerate the damage of the particle and cause cell rupture [41,42]. The damage and rupture of particles in dried samples could release more volatile and chemical compounds during the drying process.

### 3.8. Thermal Characterization (DSC) Analysis

**Figure 3:**
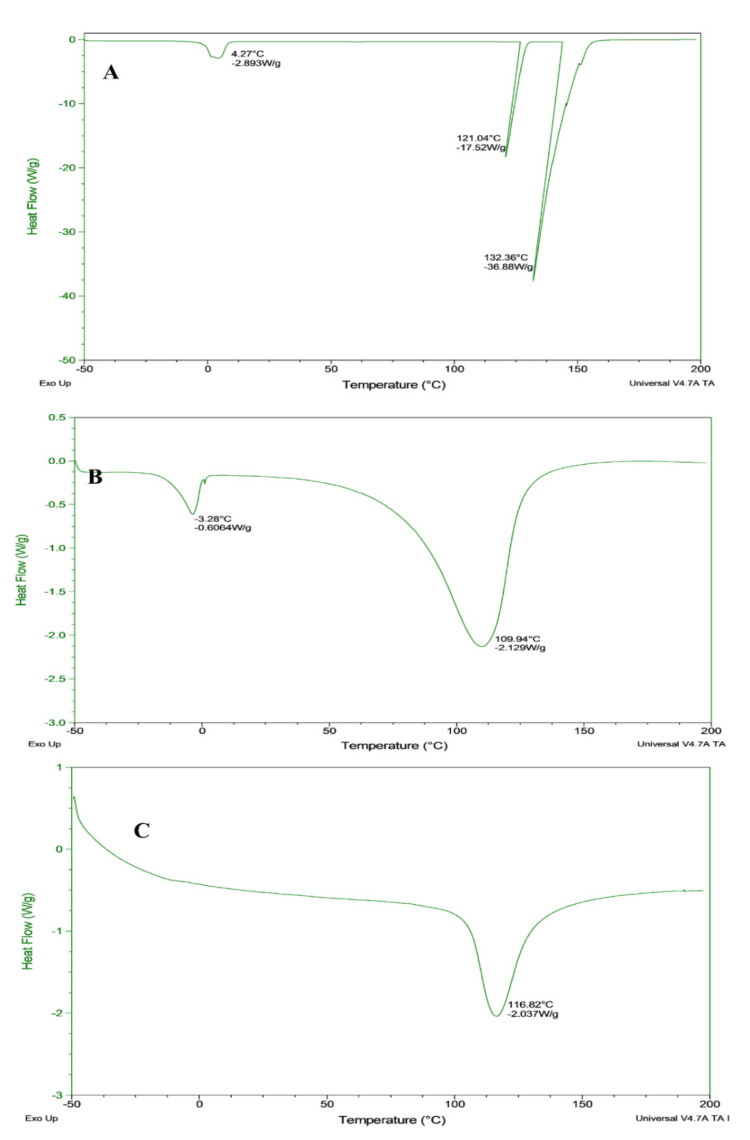
Differential scanning calorimeter of bighead carp *H. nobilis* fillets. (**A**) Raw; (**B**) oven drying; (**C**) microwave drying.

The thermal characteristics of bighead carp-treated samples, which are related to the DSC characteristics are presented in Figure 3. All of the samples exhibited a temperature range between −50 to 200 °C, with a stable flow rate of 10 °C/min. The raw fillet showed a slight thermal decomposition, which occurred at 4.27 °C. Then, the same sample showed a stable curve before the second thermal decomposition at 121 °C. The sharp thermal decomposition occurred at 132.36 °C with an intense endothermic peak.

The microwave-dried sample showed a thermal decomposition between 105 and 130 °C with an endothermic peak at a temperature of 116.8 °C. The oven-dried samples exhibited two transitions, the first at −3.28 °C with an intense endothermic peak, while the second transition was at 110 °C. The dried and raw fillets showed an endothermic response, which indicated that most of the protein and lipid contents were considerably exposed and unfolded.

The current study revealed that during drying, exothermic peaks were not found in the processed samples or the overshadowing might occur by the overlapping endothermic events. Comparing the results obtained in this study with the data investigated by Marten et al. [43], the heating rate influenced both the transition temperature and transition enthalpy in the processed samples. The endothermic transition might be assigned to the denaturation of myofibrillar and sarcoplasmic protein [44].

However, T_d_ showed a difference in the thermal decomposition temperature. In this case, (T_d_) in microwave-dried fillets was slightly higher compared with the oven-dried fillets due to the irradiation in the microwave drying technique. In this study, the different values of thermal decomposition refer to the drying techniques with various responses of protein during the process, which show the ability and potential application of fish fillets during the heating application.

### 3.9. Color Evaluation

The presented data of the color measuring assessment (L*, a*, b*) are shown in Table 1. The L* values for (oven-microwave and raw fish) samples were 72.02, 69.54, and 77.90, respectively. However, the (a*) value showed data from (2.84 to 5.28), while the (b*) value ranged from (16.15 to 20.89). The oven-dried fillets tend to have a high L* value (more lightness) compared with the microwave-dried fillets, but with a low value of a* (less redness).

The L* and a* values recorded significant differences (*p* < 0.05) among the investigated samples. The raw fillets showed the highest value of (L*) (much lightness) and less redness (a*) and yellowness (b*) compared with the other treated fillets. Color is one of the factors for the detection of good processed fish products and the change in fish color could be due to unfavorable conditions. In addition, the oxidation of lipids during the drying process may cause the browning of fish processed fillets, in reaction with protein [45]. The inhibition of the browning factor is one of the most important protocols in order to enhance the quality of dried fish. The L* values (lightness) are usually applied to indicate the browning during food drying techniques. The highest browning fillet was the microwave-dried fillet indicated by a lower L* value, followed by the oven-dried fillet, and both were different from the raw fish fillets.

This study was slightly similar to the previous study on fish sardines subjected to different drying methods [46]. The total color differences (ΔE) rated from 4.16 to 6.57 and the difference in color characteristics were related to the different drying processes and chemical reactions during the heating treatment, which could be based on the type of fish or harvesting place. However, the feeding of fish might be influenced by the total color differences.

## 4. Conclusions

In conclusion, the present study generally revealed the significant influences of drying techniques on the physico-chemical, nutritional, and thermal stability of bighead carp fillets. This work showed different values of amino acids, volatile compounds, and fatty acids. In addition, no significant difference was observed in the minerals composition during the drying process. Moreover, most of the volatile compounds that existed in processed fillets were aldehydes and organic acids. The drying process showed sufficient amounts of PUFAs and improved the quality of polyunsaturated fatty acids and essential amino acids in processed fillets. The present study provides a sufficient analysis in nutritional, thermal, and morphological properties of raw and dried fillets of bighead carp. Furthermore, this study proposes effective drying techniques for freshwater fish fillets in order to enhance their quality when they result as a dry product.

## Figures and Tables

**Table 1:** Chemical composition and color parameters (mean ± SD) of raw, oven, and microwave drying of bighead carp *H. nobilis* fillets.

Samples	Raw	Oven Drying	Microwave Drying
Moisture (%)	77.69 ± 0.88 ^a^	24.19 ± 0.91 ^b^	23.92 ± 0.86 ^b^
Ash ^1^	1.06 ± 0.05 ^c^	3.48 ± 0.06 ^b^	3.62 ± 0.07 ^a^
Fat ^1^	1.07 ± 0.12 ^b^	4.10 ± 0.16 ^a^	4.45 ± 0.15 ^a^
Protein ^1^	17.72 ± 0.25 ^b^	64.57 ± 1.47 ^a^	64.89 ± 0.58 ^a^
Ash ^2^	4.36 ± 0.07 ^b^	4.83 ± 0.06 ^a^	4.79 ± 0.16 ^a^
Fat ^2^	5.97 ± 0.82 ^a^	5.69 ± 0.22 ^a, b^	5.88 ± 0.16 ^a^
Protein ^2^	82.66 ± 1.02 ^b^	84.79 ± 0.39 ^a^	85.27 ± 0.53 ^a^
Sodium (Na) ^2^	648.57 ± 4.09 ^a^	647.63 ± 2.35 ^a^	647.85 ± 2.61 ^a^
Magnesium (Mg) ^2^	251.13 ± 3.47 ^a^	250.94 ± 4.17 ^a^	249.46 ± 1.82 ^a^
Potassium (K) ^2^	793.54 ± 5.07 ^a^	787.93 ± 3.08 ^b^	789.28 ± 2.36 ^b^
Calcium (Ca) ^2^	981.62 ± 3.25 ^a^	978.17 ± 1.73 ^b^	976.87 ± 4.07 ^b^
Iron (Fe) ^2^	21.17 ± 0.18 ^a^	20.89 ± 1.36 ^b^	20.67 ± 0.56 ^b^
Copper (Cu) ^2^	0.87 ± 0.02 ^b^	0.93 ± 0.03 ^a^	0.91 ± 0.02 ^a^
Zinc (Zn) ^2^	1.41 ± 0.12 ^b^	1.44 ± 0.09 ^a^	1.43 ± 0.34 ^a^
Ascorbic acid (C) ^2^	1.11 ± 0.00 ^a^	1.07 ± 0.07 ^a^	1.05 ± 0.02 ^a^
Thiamine (B_1_) ^2^	0.13 ± 0.01 ^a^	0.10 ± 0.01 ^a^	0.14 ± 0.02 ^a^
Riboflavin (B_2_) ^2^	0.22 ± 0.00 ^b^	0.24 ± 0.00 ^a,b^	0.27 ± 0.00 ^a^
Pyridoxine (B_6_) ^2^	0.68 ± 0.01 ^a^	0.59 ± 0.02 ^b^	0.55 ± 0.01 ^b^
Cyanocobalamin (B_12_) ^2^	0.02 ± 0.00 ^a^	0.01 ± 0.00 ^b^	0.01 ± 0.00 ^b^
Vitamin (D_3_) ^2^	0.01 ± 0.00 ^a^	0.01 ± 0.00 ^a^	0.01 ± 0.01 ^a^
Color parameters			
L*	77.90 ± 0.22 ^a^	72.02 ± 0.43 ^b^	69.54 ± 0.27 ^c^
a*	2.84 ± 0.09 ^c^	4.19 ± 0.19 ^b^	5.28 ± 0.02 ^a^
b*	16.15 ± 0.50 ^c^	19.24 ± 0.12 ^b^	20.89 ± 0.04 ^a^
ΔE	4.16 ± 0.28 ^b^	6.57 ± 0.18 ^a^	5.91 ± 0.15 ^a^

All of the values represent the mean of triplicate determinations, mean ± SD (*n* = 3). The different letters indicate that the values are significantly different (*p* ≤ 0.05). ^1^ Samples based on fresh weight (g/100 g fresh weight). ^2^ Samples based on dry weight (g/100 g dry weight). L*, a*, b*, ΔE values are international standards for color measurements. L* is the lightness value, which ranges from 0 to 100, the value a* (from green to red) and b* (from blue to yellow).

**Table 2:** Amino acid compositions of raw, oven, and microwave drying of bighead carp *H. nobilis* fillets.

Samples	Raw	Oven Drying	Microwave Drying
Essential amino acids (EAAs)			
Histidine	1.98 ± 0.11 ^b^	2.31 ± 0.14 ^a^	2.16 ± 0.21 ^a, b^
Threonine	3.43 ± 0.03 ^a^	3.32 ± 0.04 ^b^	3.24 ± 0.06 ^b^
Valine	5.46 ± 0.04 ^a^	5.29 ± 0.08 ^b^	5.41 ± 0.02 ^a^
Methionine	3.38 ± 0.03 ^c^	3.65 ± 0.06 ^a^	3.52 ± 0.04 ^b^
Phenylalanine	4.71 ± 0.05 ^a^	4.42 ± 0.06 ^b^	4.64 ± 0.03 ^a^
Isoleucine	4.67 ± 0.04 ^a^	4.39 ± 0.04 ^c^	4.55 ± 0.05 ^b^
Leucine	7.62 ± 0.02 ^b^	7.51 ± 0.03 ^c^	7.68 ± 0.02 ^a^
Lysine	8.34 ± 0.05 ^c^	8.48 ± 0.03 ^b^	8.63 ± 0.02 ^a^
Non-essential amino acids (NAAs)			
Tyrosine	2.72 ± 0.07 ^a^	2.54 ± 0.02 ^b^	2.48 ± 0.01 ^b^
Cystenie	0.34 ± 0.01 ^c^	0.47 ± 0.02 ^a^	0.41 ± 0.02 ^b^
Aspartic acid	9.61 ± 0.06 ^b^	9.76 ± 0.04 ^a^	9.85 ± 0.06 ^a^
Glutamic acid	16.07 ± 0.03 ^b^	16.36 ± 0.05 ^a^	15.80 ± 0.04 ^c^
Serine	3.59 ± 0.04 ^a^	3.38 ± 0.03 ^b^	3.28 ± 0.03 ^c^
Glycine	3.77 ± 0.03 ^c^	4.07 ± 0.04 ^b^	4.19 ± 0.03 ^a^
Arginine	5.30 ± 0.03 ^a^	5.07 ± 0.04 ^b^	4.98 ± 0.07 ^b^
Proline	2.69 ± 0.06 ^b^	2.49 ± 0.06 ^c^	2.86 ± 0.08 ^a^
Alanine	5.13 ± 0.04 ^b^	5.27 ± 0.03 ^a^	5.06 ± 0.04 ^b^
TEAA	39.65 ± 0.04 ^b^	39.71 ± 0.16 ^a, b^	39.83 ± 0.24 ^a^
TNAA	49.25 ± 0.06 ^b^	49.41 ± 0.10 ^a^	49.16 ± 0.07 ^c^
TAA	88.90 ± 0.08 ^b^	89.11 ± 0.20 ^a^	88.99 ± 0.16 ^b^

All of the values represent the mean of triplicate determinations, mean ± SD (*n* = 3). Different letters indicate that the values are significantly different (*p* ≤ 0.05). g/100 g protein sample.

**Table 3:** Volatile compounds of raw, oven, and microwave drying of bighead carp *H. nobilis* fillets.

Samples	Raw (%)	Oven Drying (%)	Microwave Drying (%)
Methylamine, N, N-dimethyl-	ND	ND	ND
Oxirane, 2,3-diethyl-	ND	ND	ND
Hexanal	0.15	1.01	1.13
Undecane	1.55	0.86	0.68
2-Hydroxymandelic acid, ethylester, di-TMS	ND	1.46	1.07
1-Penten-3-ol	0.93	ND	0.69
Cyclohexene, 1-methyl-4-(1-methylethenyl)-,(S)-	0.97	ND	0.57
Tetradecane	ND	0.98	0.63
1-Pentanol	0.79	ND	ND
Acetoin	0.68	ND	1.92
1-Hexanol	2.94	ND	ND
Nonanal	ND	ND	0.71
1-Octen-3-ol	ND	6.7	ND
Pentasiloxane, dodecamethyl-	ND	ND	2.97
2,3-Butanediol	ND	ND	ND
Propanoic acid, 2-methyl-	ND	8.97	5.24
Cyclohexanol,5-methyl-2-(1 methylethyl)-,(1à,2á,5à)-(ñ)-	8.47	5.39	4.62
Nonadecane	14.37	5.75	5.02
Nonanoic acid	ND	1.73	0.91
1,4,7,10,13,16-Hexaoxacyclooctadecane	ND	ND	ND
2-Butanol, 3-methyl-	5.92	3.21	7.26
Diazene, dimethyl-	ND	1.42	ND
2-Butanone	ND	4.03	ND
2,7-Octadiene-1,6-diol, 2,6-dimethyl-, (E)-	ND	1.37	ND
3-Heptanone, 6-methyl-	ND	2.6	ND
Nonanone	ND	2.21	3.08
2-Oxo-4-phenyl-6-(4-chlorophenyl)-1,2-dihydropyrimidine	8.12	4.87	1.08
Acetonitrile	ND	0.35	ND
Butanoic acid, 3-methyl-	ND	15.08	7.38
Butanoic acid, 2-(aminooxy)-	0.47	4.14	0.9
Acetic acid, [(aminocarbonyl)]amino]oxo	ND	ND	0.89
Vinyl butyrate	ND	ND	1.7
Acetic acid	ND	1.05	4.73
Heptadecane	ND	ND	21.61
Hexaethylene glycol	ND	ND	5.3
Ethyne, fluoro-	0.96	2.08	ND
p-Trimethylsilyloxyphenyl-bis(trimethylsilyloxy) ethane	3.40	ND	ND
1,3,6-Octatriene,3,7-dimethyl-, (Z)-	0.5	ND	ND
Cetene	0.44	0.9	ND
Acetaldehyde, tetramer	3.35	ND	ND
2-Butenoic acid, 2 methoxy-, methyl esters, (Z)-	1.25	0.19	ND

All of the values given are means of two determinations of the mean ± standard deviation. ND means not detected.

**Table 4:** Fatty acids composition (%) of raw, oven, and microwave drying of bighead carp *H. nobilis* fillets.

Samples	Raw	Oven Drying	Microwave Drying
Saturated fatty acids (SFAs)			
C12:0	0.32 ± 0.02 ^a^	0.24 ± 0.01 ^b^	0.19 ± 0.01 ^c^
C14:0	3.48 ± 0.03 ^a^	3.02 ± 0.07 ^b^	2.34 ± 0.03 ^c^
C15:0	1.00 ± 0.04 ^a^	0.96 ± 0.03 ^a^	0.67 ± 0.03 ^b^
C16:0	18.74 ± 0.09 ^b^	18.50 ± 0.18 ^b^	19.16 ± 0.08 ^a^
C17:0	1.12 ± 0.06 ^b^	1.14 ± 0.08 ^b^	2.33 ± 0.07 ^a^
C18:0	6.44 ± 0.10 ^a^	4.60 ± 0.07 ^c^	4.91 ± 0.05 ^b^
C20:0	0.22 ± 0.03 ^c^	0.31 ± 0.02 ^b^	0.38 ± 0.03 ^a^
C22:0	0.13 ± 0.01 ^b^	0.11 ± 0.00 ^b, c^	0.16 ± 0.01 ^a^
C24:0	1.02 ± 0.06 ^a^	0.81 ± 0.03 ^b^	0.72 ± 0.03 ^c^
Total SFAs	32.48 ± 0.13 ^a^	29.35 ± 0.04 ^a^	30.69 ± 0.06 ^a^
Monounsaturated fatty acids (MUFAs)			
C14:1	0.90 ± 0.03 ^a^	0.81 ± 0.02 ^b^	0.37 ± 0.05 ^c^
C16:1	7.15 ± 0.08 ^a^	6.87 ± 0.07 ^b^	5.65 ± 0.07 ^c^
C17:1	1.21 ± 0.02 ^a^	1.18 ± 0.01 ^a^	0.70 ± 0.10 ^b^
C18:1	27.08 ± 0.31 ^a^	25.04 ± 0.28 ^b^	25.27 ± 0.15 ^b^
C20:1	3.35 ± 0.09 ^a^	3.20 ± 0.19 ^a^	2.75 ± 0.07 ^b^
C22:1	0.61 ± 0.06 ^a^	0.49 ± 0.05 ^b^	0.21 ± 0.01 ^c^
C24:1	0.12 ± 0.02 ^a^	0.11 ± 0.01 ^a^	0.06 ± 0.00 ^b^
Total MUFAs	40.23 ± 0.46 ^a^	37.74 ± 0.26 ^b^	34.70 ± 0.22 ^c^
Polyunsaturated fatty acids (PUFAs)			
C18:2 *n*-6	7.35 ± 0.09 ^c^	8.27 ± 0.19 ^a^	7.70 ± 0.14 ^b^
C18:3 *n*-6	0.30 ± 0.01 ^b^	0.44 ± 0.04 ^a^	0.41 ± 0.03 ^a^
C18:3 *n*-3	5.02 ± 0.10 ^b^	5.96 ± 0.30 ^a^	5.30 ± 0.11 ^b^
C20:2 *n*-6	0.61 ± 0.05 ^b^	0.77 ± 0.005 ^a^	0.81 ± 0.02 ^a^
C20:3 *n*-6	0.59 ± 0.02 ^b^	0.71 ± 0.01 ^a^	0.72 ± 0.03 ^a^
C20:4 *n*-6	2.92 ± 0.04 ^c^	3.85 ± 0.20 ^b^	4.27 ± 0.19 ^a^
C20:3 *n*-3	0.61 ± 0.04 ^b^	0.87 ± 0.01 ^a^	0.91 ± 0.02 ^a^
C20:5 *n*-3 EPA	3.47 ± 0.09 ^b^	4.99 ± 0.22 ^a^	5.29 ± 0.15 ^a^
C22:6 *n*-3 DHA	5.41 ± 0.14 ^c^	5.94 ± 0.48 ^b^	7.57 ± 0.11 ^a^
Total PUFAs	26.73 ± 0.50 ^c^	31.03 ± 0.27 ^b^	32.85 ± 0.35 ^a^
PUFAs/SFAs	0.82	1.05	1.07
MUFAs/SFAs	1.23	1.28	1.13

Values represent the mean ± SD (*n* = 3). Different letters indicate that the values are significantly different (*p* ≤ 0.05). SFAs: Saturated fatty acids; MUFAs: Monounsaturated fatty acids; PUFAs: Polyunsaturated fatty acids.

## Data Availability

Not applicable.
